# Preconditioning Serum Levels of Endothelial Cell-Derived Molecules and the Risk of Posttransplant Complications in Patients Treated with Allogeneic Stem Cell Transplantation 

**DOI:** 10.1155/2014/404096

**Published:** 2014-10-08

**Authors:** Roald Lindås, Tor Henrik Andersson Tvedt, Kimberley Joanne Hatfield, Håkon Reikvam, Øystein Bruserud

**Affiliations:** ^1^Section for Hematology, Department of Medicine, Haukeland University Hospital, 5021 Bergen, Norway; ^2^Section for Hematology, Department of Clinical Science, University of Bergen, 5021 Bergen, Norway

## Abstract

Endothelial cells are involved in the pathogenesis of acute graft-versus-host disease (GVHD) after allogeneic stem cell transplantation. These cells express several molecules that can be detected as biologically active soluble forms; serum levels of these molecules may thereby reflect the functional status of endothelial cells. Furthermore, acute GVHD is an inflammatory reaction and endothelial cells function as local regulators of inflammation. We therefore investigated whether differences in preconditioning/pretransplant serum levels of endothelium-expressed molecules (i.e., endocan, vascular cell adhesion molecule 1 (VCAM-1), and E-selectin) were associated with a risk of posttransplant GVHD. Our study should be regarded as a population-based study of consecutive and thereby unselected patients (*n* = 56). Analysis of this pretreatment endothelium biomarker profile by unsupervised hierarchical clustering identified a subset of patients with increased early nonrelapse mortality. Furthermore, low endocan levels were significantly associated with acute GVHD in the liver and gastrointestinal tract, whereas high VCAM-1 levels were associated with acute GVHD in the skin only. Our study suggests that the preconditioning/pretransplant status of endothelial cells (possibly through altered trafficking of immunocompetent cells) is important for the risk and the organ involvement of later acute GVHD.

## 1. Introduction

Allogeneic hematopoietic stem cell transplantation has a strong antileukemic effect [1, 2] but is also associated with a relatively high risk of serious posttransplant complications, for example, graft-versus-host disease (GVHD) [3]. Endothelial cells and endothelial cell damage seem to be involved in the development of several posttransplant complications, including GVHD [4–6]. Furthermore, endothelial cell adhesion molecules are highly expressed after allotransplantation especially in GVHD-affected tissue, and soluble E-selectin (CD62E) serum levels are increased during acute GVHD whereas levels of intercellular adhesion molecule-1 (ICAM-1) and vascular cell adhesion molecule 1 (VCAM-1) are only increased in chronic GVHD [6–8]. Levels of circulating endothelial cells can also be a marker of conditioning-induced endothelial damage [9, 10]. All these observations are consistent with the hypothesis that endothelial cells are important in GVHD development. Finally, endocan is a proteoglycan expressed by endothelial cells; a soluble form is detected in serum [11] and the serum levels can be altered by infections, trauma, and malignancies as well as nonmalignant disorders [12–18]. The levels are also altered by antileukemic chemotherapy [12, 13], but serum endocan levels in allotransplant recipients have not been investigated.

Previous studies have demonstrated that the preconditioning/pretransplant clinical status is important for the risk of developing severe posttransplant complications [19, 20], and we therefore investigated whether the pretransplant serum levels of endocan as well as soluble adhesion molecules derived from endothelial cells (E-selectin and VCAM-1) are associated with the development of posttransplant acute GVHD. In contrast to the previous studies mentioned above we did not investigate the possible diagnostic or prognostic use of endothelial biomarkers during GVHD; we examined the possible associations between pretreatment levels and development of posttransplant complications. The three biomarkers were selected for our study because (i) they either are endothelial-specific (E-selectin and endocan) or are expressed only by a limited number of cells and mainly immunocompetent cells in addition to the endothelial cells (VCAM-1) and (ii) all three are important for leukocyte migration across the vessel wall and may therefore be involved in the pathogenesis of acute GVHD [21, 22].

## 2. Methods

### 2.1. Patients and Healthy Controls

The studies were approved by the Regional Ethics Committee III, University of Bergen, Norway. Samples were collected after written informed consent. The study included 56 consecutive allotransplanted patients (Table 1) during a 77-month period, representing all adult patients from a defined geographic area (Norwegian Health Regions III, IV, and V) transplanted with a family donor; the decision to do an allotransplantation was taken by the Norwegian Advisory Board for Stem Cell Transplantation and based on national guidelines. Thus, our study should be regarded as a population-based study including an unselected consecutive group of well-characterized patients (Table 1). All patients were carefully examined for and classified with regard to comorbidity according to Sorror et al. [23]; none of the patients had liver or renal disease and the overall comorbidity score was very low (1 or 0). All except three patients were Caucasians; they all received GVHD prophylaxis with cyclosporin A plus methotrexate and all except two aplastic anemia patients were transplanted with granulocyte colony-stimulating factor (G-CSF) mobilized peripheral blood stem cells. Sinusoidal obstruction syndrome was not diagnosed in any patient. Neutrophil reconstitution was defined as three consecutive days with neutrophil counts of ≥0.2/0.5 × 10^9^/L and platelet reconstitution as platelet counts ≥20/50 × 10^9^/L for at least 3 consecutive days. Capillary leak syndrome was defined as at least 10% weight gain during 24 hours despite diuretic therapy. All samples were collected before start of conditioning therapy (median 19 days before, range 3–56 days).

GVHD was diagnosed according to generally accepted criteria [24, 25]. Briefly, the diagnosis of acute GVHD was generally based on careful clinical evaluation and additional skin biopsies for patients with skin involvement alone. The diagnosis of acute GVHD for the patients with liver and/or gastrointestinal involvement was also based on careful clinical evaluation and additional biopsies (including 8 with skin biopsies and 5 with biopsies from the gastrointestinal tract) except for one patient with liver involvement where acute GVHD was a clinical diagnosis.

The controls included 19 Caucasians (median age 42 years, range 23–57 years; 10 females and 9 males).

### 2.2. Analysis of Serum Adhesion Molecule Levels

Venous blood was collected (BD Vacutainer SST Serum Separation Tubes, Becton-Dickenson; Franklin Lakes, NJ, USA) and allowed to coagulate for 120 minutes at room temperature before centrifugation (1300 g for 10 minutes) and serum collection. Serum was immediately frozen and stored at −80°C until analyzed. Average storage time for patients was 30 months and for controls 25 months. Endocan levels were determined by ELISA analyses (Lunginnov, Pasteur Institute, Lille, France). E-selectin (CD62E) and VCAM-1 were analyzed by Luminex analyses (R&D Systems; Abingdon, UK). All analyses were performed in duplicate strictly according to the manufacturers' instructions. Due to technical reasons VCAM-1 and E-selectin were analyzed only for 50 consecutive/unselected patients.

### 2.3. Statistical and Bioinformatical Analyses

Bioinformatical analyses were performed using the J-Express (MolMine AS; Bergen, Norway) [26]. For hierarchical clustering all values were median variance standardized and log(2) transformed. The complete linkage was used as linkage method, and for distance measured the Euclidean correlation was used. Additional statistical analyses were performed using the Statistical Package for the Social Sciences version 15.0 (SPSS Inc., Chicago, IL, USA) and GraphPad Prism 4 (Graph Pad Software, Inc., San Diego, CA, USA). Pearson correlation for bivariate samples was used for correlation analyses and the Mann-Whitney *U* test and chi-square test were used to compare different groups. Differences were regarded as statistically significant when *P* values < 0.05.

## 3. Results

### 3.1. Pretransplant Serum Levels of Endothelium-Derived Adhesion Molecules Show a Wide Variation

**Figure 1:**
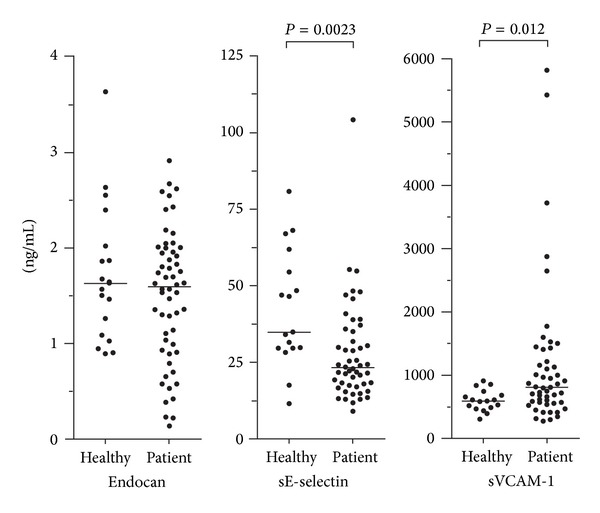
Serum levels of soluble adhesion molecules derived from endothelial cells (endocan, E-selectin, and VCAM-1) in healthy controls (healthy) and in consecutive/unselected patients prior to pretransplant conditioning therapy and allogeneic stem cell transplantation. A total of 56 patients were studied (endocan), but VCAM and E-selectin levels were available only for 50 consecutive/unselected patients.

We compared the preconditioning/pretransplant serum endocan levels for 56 consecutive patients with the levels of 19 healthy controls (Figure 1); the endocan levels did not differ significantly whereas the patients showed significantly decreased sE-selectin (Mann-Whitney *U* test, *n* = 50, *P* = 0.0023) and increased sVCAM-1 levels (*n* = 50, *P* = 0.012). Furthermore, all acute leukemia patients had undergone intensive chemotherapy and had normal bone marrow blast counts at the time of pretransplant sampling, but the hematological reconstitution varied. Six patients had peripheral blood neutrophil counts below 1 × 10^9^/L. With regard to platelet reconstitution 6 patients had pretransplant peripheral blood platelets <100 × 10^9^/L, 14 patients had 100–140 × 10^9^/L, 31 patients had normal counts, and 5 patients had preconditioning/pretransplant counts above the upper normal limit.

### 3.2. The Preconditioning Serum Profile of Endothelium-Derived Molecules Identifies a Patient Subset with Increased Frequency of Acute GVHD

**Figure 2:**
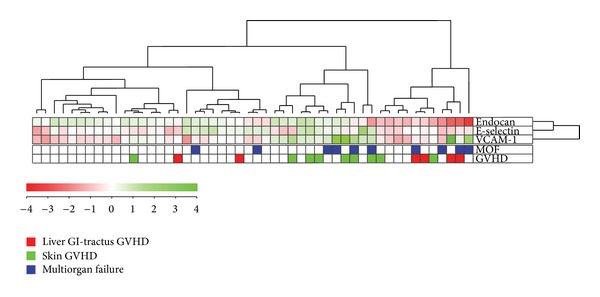
Differences in the serum profile of soluble adhesion molecules derived from endothelial cells (endocan, E-selectin, and VCAM-1) are associated with the frequency of posttransplant acute GVHD. Serum levels of all three endothelium-derived soluble adhesion molecules were determined for 50 consecutive/unselected patients (only those patients where the levels of all three mediators were available) prior to pretransplant conditioning therapy. An unsupervised hierarchical cluster analysis was performed and two major patient subsets were then identified, that is, the right 23 and the left 27 patients. The observation of acute GVHD is indicated in the right part of the figure; green squares indicate skin GVHD alone and red squares indicate that the patients had gastrointestinal or liver affection eventually in combination with skin GVHD. Early (i.e., within 4 weeks of posttransplant) nonrelapse death due to multiorgan failure (MOF) or early acute GVHD grade 4 with later death due to multiorgan failure is also indicated in the figure.

We investigated the possible association between the preconditioning endothelial biomarker profile and acute GVHD by using unsupervised hierarchical clustering analysis and including 50 consecutive/unselected patients (Figure 2). Two main patient subsets were identified: one including 23 patients (the right subset in Figure 2) and the other including 27 patients. VCAM-1 was the only biomarker that differed significantly between these two subsets (Table 2; Mann-Whitney *U* test, *P* = 0.0048). Thus, serum VCAM-1 levels have a major impact on the identification of these two patient subsets. Furthermore, acute GVHD was observed in 12 of the 23 patients in the right subset but only for three of the 27 patients in the left subset (Figure 2, chi-square test, *P* = 0.0016). Our analysis also identified a small subset, that is, the 10 patients in the outer left cluster, without acute GVHD. Finally, the right main cluster showed an increased overall frequency of early death due to multiorgan failure (Figure 2, chi-square test, *P* = 0.007). There was also a difference in platelet reconstitution between these two main subsets with regard to time until peripheral blood platelet count >20 × 10^9^/L (Table 2, *P* = 0.032) and >50 × 10^9^/L (*P* = 0.028), whereas there was only a trend for different time until neutrophils >0.5 × 10^9^/L (*P* = 0.052).

**Figure 3:**
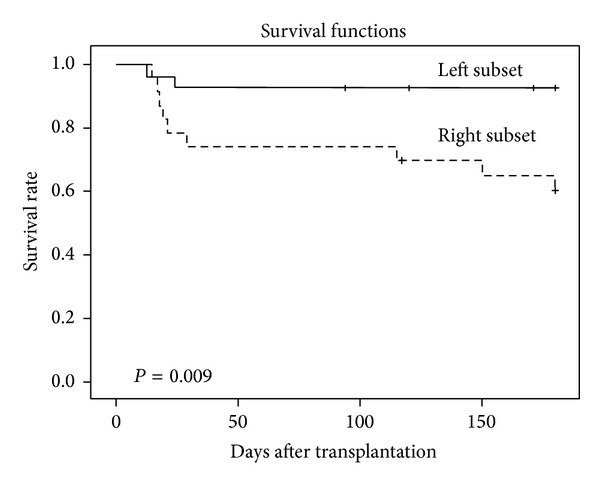
The association between serum adhesion molecule profile and survival. The serum profile of endothelium-derived soluble adhesion molecules identifies two major patient subsets that differ in survival. Based on the analysis presented in Figure 2 we identified two main patient clusters. We did a Kaplan-Mayer analysis to compare the survival between these two subsets, and a significant difference was then detected between the two groups. This difference was caused by nonrelapse mortality.

The two main clusters identified in Figure 2 did not differ with regard to age, gender distribution, diagnosis, disease stage, organ affection or severity of acute GVHD, and maximal weight gain during the first four weeks after start of conditioning therapy (data not shown). Thirty-six of the 50 patients included in the cluster analysis were followed for at least 1 year after transplantation. There was a significant difference in survival between these two main subsets (Figure 3), but the two clusters showed no significant difference in the frequencies of chronic GVHD and leukemia relapse (data not shown).

### 3.3. Patients with Acute GVHD of the Liver and Gastrointestinal Tract Show Low Serum Endocan Levels

Serum endocan levels prior to conditioning treatment were determined for 56 consecutive patients and showed a wide variation (Figure 1; median 1.57 ng/mL, range 0.15–2.91 ng/mL) without any significant associations with age, gender, diagnosis, relapse versus first diagnosis, or peripheral blood platelet count at the time of sampling (data not shown).

**Figure 4:**
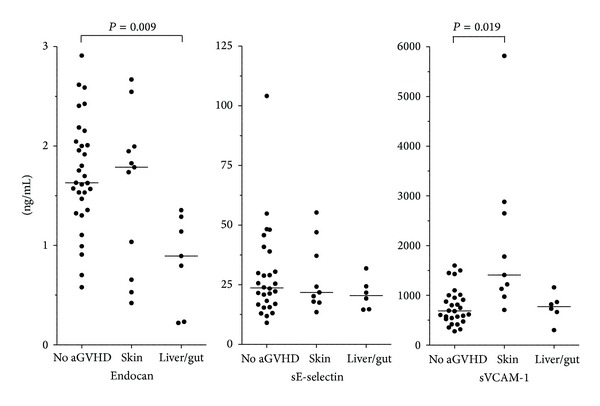
Differences in the serum profile of soluble adhesion molecules derived from endothelial cells (endocan, E-selectin, and VCAM-1) are associated with posttransplant acute GVHD. Serum levels of endothelium-derived soluble adhesion molecules were compared for patients without acute GVHD, patients with skin GVHD alone, and patients with liver and/or gastrointestinal affection during acute GVHD. Serum levels of endocan were available for all 56 patients (11 with acute GVHD in the skin and 7 with liver/gut involvement); for VCAM-1 and E-selectin serum levels were available only for 50 consecutive/unselected patients (9 with acute GVHD in the skin only and 6 with liver/gut involvement). The statistically significant differences (Mann-Whitney *U* test) are indicated in the figure.

We then compared patients with preconditioning serum levels above and below the median level of 1.57 ng/mL. The overall frequency and severity of acute GVHD did not differ between these two groups, but all seven patients with acute GVHD affecting the liver and/or gastrointestinal tract showed serum endocan concentrations below the median level (Figure 4). Thus, there is a statistically significant association between low preconditioning/pretransplant serum endocan levels and later organ involvement in acute GVHD. This difference was statistically significant when comparing the liver/gastrointestinal GVHD patients both with all other patients (Mann-Whitney *U* test, *P* = 0.004) and with the subset of patients without GVHD (Figure 4, *P* = 0.009). The maximal weight variation during the first 4 weeks after start of conditioning did not differ between patients with serum endocan levels above (median weight variation 5.5 kg, variation range −6 to 18 kg) and below (median 5.6 kg, range −6 to 20 kg) the median level. Preconditioning serum endocan levels showed no correlation with (i) the corresponding levels of VCAM-1 (*P* = 0.43) and E-selectin (*P* = 0.28) or (ii) time until neutrophil/platelet reconstitution (data not shown).

A consecutive subset of 42 patients were observed for at least 12 months after stem cell transplantation; for these patients preconditioning serum endocan levels showed no association with the later development of chronic GVHD (data not shown).

### 3.4. Patients with Acute GVHD Only Affecting the Skin Show High Serum VCAM-1 prior to Therapy

Serum VCAM-1 levels were determined before start of conditioning therapy for 50 unselected patients. We observed skin GVHD alone in 9 patients and 8 of them showed serum VCAM-1 levels higher than the median level for all 50 patients (Figure 4). This difference was statistically significant both when comparing the skin-alone GVHD patients with all other patients (Mann-Whitney *U* test, *P* = 0.011) and when comparing them with the subset of patients without GVHD (Figure 4, *P* = 0.019). In contrast, serum VCAM-1 levels did not show any associations with the frequency of gastrointestinal and liver GVHD, time until neutrophil/platelet reconstitution, or maximal weight variation during the first 4 weeks after start of conditioning therapy. The preconditioning serum VCAM-1 and E-selectin levels showed no significant correlation (*P* = 0.45). Finally, a consecutive subset of 36 patients were observed at least 12 months after stem cell transplantation; for these patients the preconditioning serum VCAM-1 level showed no association with the development of chronic GVHD (data not shown).

### 3.5. Serum E-Selectin Levels before Start of Conditioning Therapy Show No Significant Associations with Later Acute GVHD

Preconditioning serum E-selectin levels showed a wide variation (Figures 1 and 4) with no associations with frequency, severity, or organ affection of acute GVHD. There was a borderline inverse correlation between preconditioning/pretransplant E-selectin level and time until neutrophils >0.2 × 10^9^/L (*P* = 0.051); this association reached statistical significance for time until neutrophils >0.5 × 10^9^/L (*P* = 0.037). Finally, we did not observe any correlation between the serum E-selectin levels and time until platelet recovery or maximal weight variation during the first 4 weeks after start of conditioning therapy (data not shown).

## 4. Discussion

Few previous studies have investigated the possible importance of preconditioning/pretransplant serum mediator levels as prognostic biomarkers in allogeneic stem cell transplantation; previous studies have mainly focused on single endothelial biomarkers as diagnostic or prognostic tools in patients with manifest acute GVHD rather than the possible use or biomarker profiles in the pretransplant evaluation of allotransplant recipients. Endothelial cells are important regulators of immunocompetent cell trafficking, and soluble forms of endothelium-derived molecules may thereby alter cell trafficking or reflect the functional status of endothelial cells [9, 10, 27]. Our results suggest that even the endothelial cell status prior to conditioning is important for the risk of acute GVHD.

The current treatment of acute GVHD is based on general immunosuppression [28]. However, therapeutic targeting of immunocompetent cell trafficking by blocking the CCR5 chemokine receptor has also been tried, and patients receiving this prophylaxis only had skin but not gut or liver GVHD [29]. These results together with our present observations are thus consistent with the hypothesis that organ-dependent differences in endothelial cell phenotype influence the organ involvement in acute GVHD [30].

Pretransplant characteristics are important for the risk of posttransplant acute GVHD, for example, advanced disease, the conditioning regimens, and previous viral infections [20, 31], and even differences in the pretransplant serum cytokine profile seem to be associated with the risk of GVHD [19]. The serum levels of soluble adhesion molecules derived from endothelial cells may serve as additional markers of endothelial damage or functional status [6, 7, 32–34]. Several other soluble mediators have been examined previously, for example, thrombomodulin and von Willebrand factor [9], but as discussed in detail previously [9] they have several limitations and their systemic levels depend on various host factors including renal function, pharmacotherapy, liver disease, viral infections, or other diseases like hypertension and diabetes. In this context we studied the preconditioning/pretransplant serum profile of endothelium-derived soluble molecule for a population-based, well-characterized, and consecutive group of recipients.

Few previous studies have investigated associations between pretransplant biomarker levels and the posttransplant clinical course after allotransplantation [26, 32–34]. Firstly, the risk of serious complications early after allotransplantation is associated with the preconditioning serum profile of immunoregulatory and angioregulatory mediators [19]. Secondly, an association between serum levels of angioregulatory angiopoietin-2/Tie2, and relapse-free survival was described for a selected group of high-risk patients with only 17 out of 90 patients being in complete remission prior to transplantation [35, 36]. These patients are thus not comparable to our unselected patients. Thirdly, in a pediatric study Porkholm et al. [37] described that pretransplant high angiopoietin-2 plasma levels were associated with increased risk of gastrointestinal GVHD and nonrelapse mortality, but in contrast to our study these authors examined the levels at day 0* after conditioning*. Finally, endocan and soluble adhesion molecules were not examined in previous studies that focused on endothelial markers as prognostic parameters with regard to GVHD response to steroid treatment, and these authors did not detect any differences in pretransplant biomarkers between patients with or without later acute GVHD [35–37]. Thus, our present study is the only one to suggest an association between the endothelial cell status prior to conditioning therapy and risk of later acute GVHD.

Our serum samples were prepared by a standardized procedure and the measured biomarker levels will include the levels of biologically active free molecules that are released after proteolysis or by secretion [7, 32, 33]. However, we cannot exclude the possibility that endothelium-derived microparticles are present in our samples and contribute to the measured biomarker levels. Microparticles can be removed by ultracentrifugation [38, 39], that is, a method usually not available at routine clinical laboratories. Our intention was to investigate biomarker levels in samples that can be prepared as a part of routine clinical evaluation of these patients [7], and for that reason we used standard serum samples.

Gastrointestinal and liver GVHD were associated with low endocan levels whereas acute GVHD only involving the skin showed increased VCAM-1 levels (Figure 4); a possible explanation for this is organ-dependent differences of the endothelial cell phenotype for the involved organs. Proteomic studies have clearly demonstrated that the endothelial cell phenotype differs between organs; this is probably because the tissue microenvironment surrounding the blood vessels controls the phenotype [40, 41]. This influence of the microenvironment has also been documented in experimental studies [42], and the differences are so extensive that vascular targeting is even considered for directing anticancer drugs to defined compartments or organs [43]. Only a minor part of this proteomic heterogeneity has been characterized in detail at a molecular level, but the endothelial phenotype characterized by phage display techniques differs, for example, between skin and intestine [42] and between large blood vessels and microvessels [44]. The microenvironment-dependent variation in endothelial cell phenotype can also explain the differences in endothelium-derived biomarker levels with regard to organs involved in acute GVHD. Organ-specific variations, differences between microvessels and large vessels, and different effects on the vessel-surrounding microenvironment by various parenchymal disorders may also explain why endothelial biomarker levels in acute liver GVHD (normal VCAM-1) differ from other liver diseases, for example, cirrhosis with increased hepatic vein pressure gradients showing increased systemic VCAM-1 levels [45].

Endothelial cells contribute to the formation of vascular stem cell niches [46–48] and they are also important regulators of fluid transport across the vessel wall [49, 50]. However, the serum levels of endothelium-derived biomarkers showed no correlation with fluid retention during treatment, but associations between biomarkers and neutrophil (sE-selectin) as well as platelet reconstitution (adhesion molecule profile) could be detected.

## 5. Conclusion

We describe an association between preconditioning endothelial cell biomarkers and organ involvement in acute GVHD. Based on these observations our hypothesis is that differences in immunocompetent cell trafficking caused by differences in endothelial cell phenotype between target organs are important in the pathogenesis and thereby also for the clinical presentation of acute GVHD.

## Figures and Tables

**Table 1:** Clinical and biological characteristics of allotransplanted patients included in the study.

Age (years, median, and range)	43 (18–63)
Gender	
Males	38
Females	18
Diagnosis	
AML	33
B-ALL	11
T-ALL	4
MDS	2
CMML	2
CML	1
Myelofibrosis secondary to polycythemia vera	1
Aplastic anemia	2
Status at the time of transplantation	
First complete hematological remission	41
Second or later complete remission	8
Previous allogeneic stem cell transplantation and now complete remission	1
Detectable disease	6
Conditioning therapy	
Busulfan + cyclophosphamide	52
Total body irradiation + cyclophosphamide	1
Antithymocyte globulin + cyclophosphamide	2
BEAM (carmustine, etoposide, cytarabine, and melphalan)	1
Stem cell grafts	
Peripheral blood mobilized stem cells	54
Bone marrow	2
GVHD prophylaxis with cyclosporin A plus methotrexate	
4 methotrexate injections	34
3 methotrexate injections	21
2 methotrexate injections	1
GVHD, organ affection, and severity	
Skin	17
Liver	3
Gastrointestinal	6
Grade 1	5
Grade 2	9
Grade 3	2
Grade 4	2

**Table 2:** Unsupervised hierarchical clustering of serum soluble adhesion levels for 50 unselected patients treated with allogeneic stem cell transplantation; a comparison of the two main patient subsets including the 23 patients to the right and the 27 patients to the left in Figure 2, respectively.

Adhesion molecule—clinical parameter	Left patient subset, *n* = 27	Right patient subset, *n* = 23	*P* value
Endocan (ng/mL)	1.567 (0.908–2.452)	0.893 (0.148–2.901)	0.0516
VCAM-1 (ng/mL)	691.6 (317.3–1130.0)	1410 (300.0–5816.4)	0.0048
E-selectin (ng/mL)	25.4 (9.0–54.8)	21.7 (12.9–104.1)	0.4595
Frequency of early nonrelapse death	2/27	8/23	0.007
Time to neutrophils ≥0.2 × 10^9^/L	14.5 days (11–19 days)	15 days (10–26 days)	ns
Time to neutrophils >0.5 × 0^9^/L	16 days (11–24 days)	18 days (12–28 days)	0.052
Time to platelets >20 × 10^9^/L	12 days (9–>22 days)	16 days (12–30 days)	0.032
Time to platelets >50 × 10^9^/L	16 days (12–38 days)	25 days (13–>71 days)	0.028

The Mann-Whitney *U* test was used for statistical analyses. Results are presented as the median value and the variation range. Patients who died before reaching platelet reconstitution were not included in the statistical comparison of time until reconstitution (ns: not significant).
